# Supplementary material for: HIVprotI: an integrated web based platform for prediction and design of HIV proteins inhibitors
Source: J Cheminform. 2018 Mar 9;10:12. doi: 10.1186/s13321-018-0266-y (PMC5845081; doi:10.1186/s13321-018-0266-y)
Supplement: Supplementary file 2 — Additional file 2. Source code of HIVProtI web server. [file 13321_2018_266_MOESM2_ESM.zip › HIVprotI_Source-code/hivprotI/weka-3-6-6/doc/weka/gui/graphvisualizer/GraphVisualizer.html]

GraphVisualizer


---


|  |  |  |
| --- | --- | --- |
| **Overview** \| **Package** \| **Class** \| **Tree** \| **Deprecated** \| **Index** \| **Help** \| **Weka's home** | |  |
| **PREV CLASS**   **NEXT CLASS** | **FRAMES**    **NO FRAMES**     **All Classes** |  |
| SUMMARY: NESTED \| FIELD \| CONSTR \| METHOD | DETAIL: FIELD \| CONSTR \| METHOD |  |


---


## weka.gui.graphvisualizer Class GraphVisualizer

```
java.lang.Object
  java.awt.Component
      java.awt.Container
          javax.swing.JComponent
              javax.swing.JPanel
                  weka.gui.graphvisualizer.GraphVisualizer
```

**All Implemented Interfaces:**: java.awt.image.ImageObserver, java.awt.MenuContainer, java.io.Serializable, javax.accessibility.Accessible, GraphConstants, LayoutCompleteEventListener

---

``` public class GraphVisualizer extends javax.swing.JPanel implements GraphConstants, LayoutCompleteEventListener ```

This class displays the graph we want to visualize. It should
be sufficient to use only this class in weka.gui.graphvisulizer
package to visualize a graph. The description of a graph should
be provided as a string argument using readBIF or readDOT method
in either XMLBIF03 or DOT format. Alternatively, an InputStream
in XMLBIF03 can also be provided to another variation of readBIF.
It would be necessary in case input is in DOT format to call the
layoutGraph() method to display the graph correctly after the call
to readDOT. It is also necessary to do so if readBIF is called and
the graph description doesn't have x y positions for nodes.

The graph's data is held in two FastVectors, nodes are stored as
objects of GraphNode class and edges as objects of GraphEdge class.

The graph is displayed by positioning and drawing each node
according to its x y position and then drawing all the edges coming
out of it give by its edges[][] array, the arrow heads are ofcourse
marked in the opposite(ie original direction) or both directions if
the edge is reversed or is in both directions. The graph is centered
if it is smaller than it's display area. The edges are drawn from the
bottom of the current node to the top of the node given by edges[][]
array in GraphNode class, to avoid edges crossing over other nodes.
This might need to be changed if another layout engine is added or
the current Hierarchical engine is updated to avoid such crossings
over nodes.

**Version:**
:   $Revision: 7059 $

**Author:**
:   Ashraf M. Kibriya (amk14@cs.waikato.ac.nz)

**See Also:**: Serialized Form

---

**Nested Class Summary**

**Nested classes/interfaces inherited from class javax.swing.JComponent**

`javax.swing.JComponent.AccessibleJComponent`

**Nested classes/interfaces inherited from class java.awt.Component**

`java.awt.Component.BaselineResizeBehavior`


**Field Summary**

**Fields inherited from class javax.swing.JComponent**

`TOOL_TIP_TEXT_KEY, UNDEFINED_CONDITION, WHEN_ANCESTOR_OF_FOCUSED_COMPONENT, WHEN_FOCUSED, WHEN_IN_FOCUSED_WINDOW`

**Fields inherited from class java.awt.Component**

`BOTTOM_ALIGNMENT, CENTER_ALIGNMENT, LEFT_ALIGNMENT, RIGHT_ALIGNMENT, TOP_ALIGNMENT`

**Fields inherited from interface weka.gui.graphvisualizer.GraphConstants**

`DIRECTED, DOUBLE, NORMAL, PLURAL_DUMMY, REVERSED, SINGULAR_DUMMY`

**Fields inherited from interface java.awt.image.ImageObserver**

`ABORT, ALLBITS, ERROR, FRAMEBITS, HEIGHT, PROPERTIES, SOMEBITS, WIDTH`


**Constructor Summary**

`GraphVisualizer()`
  
          Constructor  
Sets up the gui and initializes all the other previously
uninitialized variables.


| **Method Summary** | |
| --- | --- |
| `void` | `layoutCompleted(LayoutCompleteEvent le)`             This method is an implementation for LayoutCompleteEventListener class. |
| `void` | `layoutGraph()`             This method lays out the graph by calling the LayoutEngine's layoutGraph() method. |
| `static void` | `main(java.lang.String[] args)`             Main method to load a text file with the description of a graph from the command line |
| `void` | `readBIF(java.io.InputStream instream)`             BIF reader  Reads a graph description in XMLBIF03 from an InputStrem |
| `void` | `readBIF(java.lang.String instring)`             BIF reader  Reads a graph description in XMLBIF03 from a string |
| `void` | `readDOT(java.io.Reader input)`             Dot reader  Reads a graph description in DOT format from a string |

**Methods inherited from class javax.swing.JPanel**

`getAccessibleContext, getUI, getUIClassID, setUI, updateUI`

**Methods inherited from class javax.swing.JComponent**

`addAncestorListener, addNotify, addVetoableChangeListener, computeVisibleRect, contains, createToolTip, disable, enable, firePropertyChange, firePropertyChange, firePropertyChange, getActionForKeyStroke, getActionMap, getAlignmentX, getAlignmentY, getAncestorListeners, getAutoscrolls, getBaseline, getBaselineResizeBehavior, getBorder, getBounds, getClientProperty, getComponentPopupMenu, getConditionForKeyStroke, getDebugGraphicsOptions, getDefaultLocale, getFontMetrics, getGraphics, getHeight, getInheritsPopupMenu, getInputMap, getInputMap, getInputVerifier, getInsets, getInsets, getListeners, getLocation, getMaximumSize, getMinimumSize, getNextFocusableComponent, getPopupLocation, getPreferredSize, getRegisteredKeyStrokes, getRootPane, getSize, getToolTipLocation, getToolTipText, getToolTipText, getTopLevelAncestor, getTransferHandler, getVerifyInputWhenFocusTarget, getVetoableChangeListeners, getVisibleRect, getWidth, getX, getY, grabFocus, isDoubleBuffered, isLightweightComponent, isManagingFocus, isOpaque, isOptimizedDrawingEnabled, isPaintingForPrint, isPaintingTile, isRequestFocusEnabled, isValidateRoot, paint, paintImmediately, paintImmediately, print, printAll, putClientProperty, registerKeyboardAction, registerKeyboardAction, removeAncestorListener, removeNotify, removeVetoableChangeListener, repaint, repaint, requestDefaultFocus, requestFocus, requestFocus, requestFocusInWindow, resetKeyboardActions, reshape, revalidate, scrollRectToVisible, setActionMap, setAlignmentX, setAlignmentY, setAutoscrolls, setBackground, setBorder, setComponentPopupMenu, setDebugGraphicsOptions, setDefaultLocale, setDoubleBuffered, setEnabled, setFocusTraversalKeys, setFont, setForeground, setInheritsPopupMenu, setInputMap, setInputVerifier, setMaximumSize, setMinimumSize, setNextFocusableComponent, setOpaque, setPreferredSize, setRequestFocusEnabled, setToolTipText, setTransferHandler, setVerifyInputWhenFocusTarget, setVisible, unregisterKeyboardAction, update`

**Methods inherited from class java.awt.Container**

`add, add, add, add, add, addContainerListener, addPropertyChangeListener, addPropertyChangeListener, applyComponentOrientation, areFocusTraversalKeysSet, countComponents, deliverEvent, doLayout, findComponentAt, findComponentAt, getComponent, getComponentAt, getComponentAt, getComponentCount, getComponents, getComponentZOrder, getContainerListeners, getFocusTraversalKeys, getFocusTraversalPolicy, getLayout, getMousePosition, insets, invalidate, isAncestorOf, isFocusCycleRoot, isFocusCycleRoot, isFocusTraversalPolicyProvider, isFocusTraversalPolicySet, layout, list, list, locate, minimumSize, paintComponents, preferredSize, printComponents, remove, remove, removeAll, removeContainerListener, setComponentZOrder, setFocusCycleRoot, setFocusTraversalPolicy, setFocusTraversalPolicyProvider, setLayout, transferFocusBackward, transferFocusDownCycle, validate`

**Methods inherited from class java.awt.Component**

`action, add, addComponentListener, addFocusListener, addHierarchyBoundsListener, addHierarchyListener, addInputMethodListener, addKeyListener, addMouseListener, addMouseMotionListener, addMouseWheelListener, bounds, checkImage, checkImage, contains, createImage, createImage, createVolatileImage, createVolatileImage, dispatchEvent, enable, enableInputMethods, firePropertyChange, firePropertyChange, firePropertyChange, firePropertyChange, firePropertyChange, getBackground, getBounds, getColorModel, getComponentListeners, getComponentOrientation, getCursor, getDropTarget, getFocusCycleRootAncestor, getFocusListeners, getFocusTraversalKeysEnabled, getFont, getForeground, getGraphicsConfiguration, getHierarchyBoundsListeners, getHierarchyListeners, getIgnoreRepaint, getInputContext, getInputMethodListeners, getInputMethodRequests, getKeyListeners, getLocale, getLocation, getLocationOnScreen, getMouseListeners, getMouseMotionListeners, getMousePosition, getMouseWheelListeners, getName, getParent, getPeer, getPropertyChangeListeners, getPropertyChangeListeners, getSize, getToolkit, getTreeLock, gotFocus, handleEvent, hasFocus, hide, imageUpdate, inside, isBackgroundSet, isCursorSet, isDisplayable, isEnabled, isFocusable, isFocusOwner, isFocusTraversable, isFontSet, isForegroundSet, isLightweight, isMaximumSizeSet, isMinimumSizeSet, isPreferredSizeSet, isShowing, isValid, isVisible, keyDown, keyUp, list, list, list, location, lostFocus, mouseDown, mouseDrag, mouseEnter, mouseExit, mouseMove, mouseUp, move, nextFocus, paintAll, postEvent, prepareImage, prepareImage, remove, removeComponentListener, removeFocusListener, removeHierarchyBoundsListener, removeHierarchyListener, removeInputMethodListener, removeKeyListener, removeMouseListener, removeMouseMotionListener, removeMouseWheelListener, removePropertyChangeListener, removePropertyChangeListener, repaint, repaint, repaint, resize, resize, setBounds, setBounds, setComponentOrientation, setCursor, setDropTarget, setFocusable, setFocusTraversalKeysEnabled, setIgnoreRepaint, setLocale, setLocation, setLocation, setName, setSize, setSize, show, show, size, toString, transferFocus, transferFocusUpCycle`

**Methods inherited from class java.lang.Object**

`equals, getClass, hashCode, notify, notifyAll, wait, wait, wait`

**Constructor Detail**

### GraphVisualizer

```
public GraphVisualizer()
```

:   Constructor  
    Sets up the gui and initializes all the other previously
    uninitialized variables.


**Method Detail**

### layoutCompleted

```
public void layoutCompleted(LayoutCompleteEvent le)
```

:   This method is an implementation for LayoutCompleteEventListener
    class. It sets the size appropriate for m\_gp GraphPanel and
    and revalidates it's container JScrollPane once a
    LayoutCompleteEvent is received from the LayoutEngine.

    :   **Specified by:**: `layoutCompleted` in interface `LayoutCompleteEventListener`

---


### layoutGraph

```
public void layoutGraph()
```

:   This method lays out the graph by calling the
    LayoutEngine's layoutGraph() method. This method
    should be called to display the graph nicely, unless
    the input XMLBIF03 already contains some layout
    information (ie the x,y positions of nodes.

---


### readBIF

```
public void readBIF(java.lang.String instring)
             throws BIFFormatException
```

:   BIF reader  
    Reads a graph description in XMLBIF03 from a string

    :   **Throws:**: `BIFFormatException`

---


### readBIF

```
public void readBIF(java.io.InputStream instream)
             throws BIFFormatException
```

:   BIF reader  
    Reads a graph description in XMLBIF03 from an InputStrem

    :   **Throws:**: `BIFFormatException`

---


### readDOT

```
public void readDOT(java.io.Reader input)
```

:   Dot reader  
    Reads a graph description in DOT format from a string

---


### main

```
public static void main(java.lang.String[] args)
```

:   Main method to load a text file with the
    description of a graph from the command
    line


---


|  |  |  |
| --- | --- | --- |
| **Overview** \| **Package** \| **Class** \| **Tree** \| **Deprecated** \| **Index** \| **Help** \| **Weka's home** | |  |
| **PREV CLASS**   **NEXT CLASS** | **FRAMES**    **NO FRAMES**     **All Classes** |  |
| SUMMARY: NESTED \| FIELD \| CONSTR \| METHOD | DETAIL: FIELD \| CONSTR \| METHOD |  |


---
